# Dissection of ubiquitinated protein degradation by basal autophagy

**DOI:** 10.1002/1873-3468.12641

**Published:** 2017-04-18

**Authors:** Kaori Takayama, Akira Matsuura, Eisuke Itakura

**Affiliations:** ^1^ Department of NanobiologyGraduate School of Advanced Integration ScienceChiba UniversityInage‐kuJapan

**Keywords:** aggregates, autophagy, p62/sqstm1, protein degradation

## Abstract

Basal autophagy plays an essential role as a protein quality control system. Although it has been demonstrated that the loss of autophagy results in the accumulation of ubiquitin‐positive aggregates and the development of neurodegenerative diseases, the precise autophagy substrate(s) remain unclear. Here, we determined whether ubiquitinated proteins are direct substrates for basal autophagy using a fluorescent ratiometric probe for ubiquitin. We show that the degradation of polyubiquitinated proteins is not dependent on basal autophagy. Although ubiquitin‐positive aggregates are observed in autophagy knockout cultured cells, the aggregates consist of soluble and mobile polyubiquitinated proteins, which are trapped by p62 without an increase in the total amount of ubiquitinated proteins. These results suggest that ubiquitinated proteins are not major targets for basal autophagy.

## Abbreviations

**CHX**, cychloheximide

**Dox**, doxycycline

**FLIP**, fluorescence loss in photobleaching

**FRAP**, fluorescence recovery after photobleaching

**LC3**, microtubule‐associated protein 1 light chain 3

**UBA**, ubiquitin associated

Autophagy, a major protein quality control system in the cell, is a housekeeping process that is conserved from yeast to mammals. An isolation membrane (also known as a phagophore) generated in the cytosol engulfs a portion of the cytoplasm to become a double‐membrane structure, the autophagosome 1, 2, 3, 4. The outer membrane of the completed autophagosome fuses with the lysosomal membrane, exposing the enclosed cytoplasm of the autophagosome to lysosomal enzymes that degrade the contents 5. Although classical view is that autophagy is a nonselective and bulk degradation system, recent studies have highlighted the ability of autophagy to selectively eliminate specific substrates via autophagy adaptor proteins. Cytoplasmic proteins and organelles can be targeted into the autophagosome and degraded in the lysosome. In mammals, mitophagy, the degradation of mitochondria, is a well‐characterized selective autophagy system. PINK1 (PTEN‐induced putative kinase 1) accumulates on damaged mitochondria, activating Parkin E3 ligase, which functions to ubiquitinate the outer mitochondrial membrane proteins. The ubiquitinated proteins are then recognized by autophagy adaptor proteins containing ubiquitin‐binding domains 6. Autophagy adaptor proteins are essential for recognizing the ubiquitinated mitochondria to sequester them into the autophagosome 7. Xenophagy, the degradation of invading bacteria, relies on a similar system. The invading bacteria with raptured endosomal membranes are ubiquitinated, which allows recognition by the autophagy adaptor proteins 8, 9. Therefore, it is thought that ubiquitination is a critical recognition step in selective autophagy in mammals 10, 11.

A common feature of various neurodegenerative diseases is the accumulation and deposition of proteins that affect neuronal connectivity and cell death. Although most protein aggregates in neuronal diseases are positive for ubiquitin, it remains unclear how such ubiquitinated proteins accumulate 12. It has been hypothesized that autophagy is linked to neurodegenerative diseases, with critical evidence provided by the analysis of autophagy knockout (KO) mice. Neuron‐specific autophagy KO mice develop neurodegenerative disease‐like symptoms, including axonal swelling, progressive motor, and behavioral deficits, and large ubiquitin‐positive inclusion bodies in numerous neurons 13, 14. Interestingly, nutrient starvation induces autophagy in most tissues, but not in the brain 15, suggesting that low‐level constitutive autophagy (basal autophagy) in the brain has a critical role as a protein homeostasis system. Several substrates targeted by basal autophagy have been investigated 16, 17. p62/SQSTM1 is the best characterized autophagy adaptor protein containing an ubiquitin‐binding domain. Based on the facts that p62‐positive ubiquitin aggregates accumulate in autophagy‐deficient cells and that p62 binds directly to ubiquitin to link between ubiquitinated substrates and autophagosomal membranes, it is possible that ubiquitinated proteins or aggregates are also constitutively degraded by basal autophagy 18, 19. However, whether ubiquitinated proteins are incorporated into autophagosomes under basal conditions has not been directly examined.

Although ubiquitin is recycled from polyubiquitinated proteins by deubiquitination enzymes on the proteasome during the degradation of substrate, ubiquitin at the most proximal part of the ubiquitin chain can be degraded along with the substrate 20, 21. If ubiquitinated proteins are selectively engulfed by autophagosomes, it stands to reason that polyubiquitin and conjugated protein should also be degraded in the lysosome. Indeed, several studies have shown that ubiquitin is localized and degraded in the lysosome 22, 23. However, the main pathway for ubiquitin degradation remains unclear. To determine whether ubiquitinated proteins are targets for autophagy, we generated a novel fluorescent ratiometric probe for ubiquitin and employed a pulse‐chase assay to specifically measure lysosomal degradation of ubiquitin under basal conditions in mammalian cells. We revealed that almost all of the detectable ubiquitin degradation in the cell occurred by proteasomes, and that autophagy contributes little to ubiquitin turnover under basal conditions. In addition, we showed that ubiquitin‐positive aggregates in autophagy‐deficient cells are not immobile. These data led us to propose that ubiquitinated proteins and ubiquitin itself are degraded mainly by proteasomes, and the accumulation of ubiquitin‐positive aggregates in autophagy‐deficient cells may be a secondary phenotype caused by the loss of protein homeostasis.

## Materials and methods

### Plasmids

Various plasmids were constructed by inserting cDNA clones of microtubule‐associated protein 1 light chain 3 (LC3), ubiquitin, or ubiquitin mutants into pCW57.1 (Addgene plasmid 41393) or pLenti CMV GFP Puro (Addgene plasmid 17448) together with enhanced green fluorescent protein (EGFP), superfolding green fluorescent protein (sfGFP), or mCherry. The following plasmids were generated: pCW sfGFP‐Ubiquitin, pCW mCherry‐EGFP‐LC3, pCW mCherry‐EGFP‐Ubiquitin, pCW Ubiquitin^G76V^‐mCherry‐EGFP, pLenti CMV sfGFP‐p62, pLenti CMV mCherry‐Ubiquitin, and pLenti CMV mCherry‐Ubiquitin^I44A^. pCMV‐VSV‐G (Addgene plasmid 8454) and psPAX2 (Addgene plasmid 12260) were used for lentivirus production.

### Antibodies and reagents

Antibodies against LC3 (Code No. PD026), p62 (Code No. PM045), multiubiquitin (Code No. D058‐3, Clone FK2), and GFP pAb‐HRP‐DirecT (Code No. 598‐7) were purchased from MBL (Nagoya, Japan). Anti‐β‐actin (Clone No. 2F3) antibody was purchased from Wako Pure Chemical Industries (Osaka, Japan). Anti‐Hsp90 antibody was purchased from BD Bioscience (San Jose, CA, USA). Anti‐Histon H3 (clone MABI 0301) antibody was purchased from MAB Institute (Sapporo, Japan). Rabbit polyclonal antibodies against GFP and hemagglutinin (HA) have been described previously 23. Bafilomycin A_1_ (BafA, LC Laboratories, Woburn, MA, USA), MG132 (LifeSensors, Malvern, PA, USA), Torin1 (Tocris Bioscience, Ellisville, MO, USA), cycloheximide (CHX; Sigma‐Aldrich, St. Louis, MO, USA), and doxycycline (Dox; Clontech, Mountain View, CA, USA) were purchased.

### Cell culture

HeLa cells and human embryonic kidney 293FT cells were cultured in Dulbecco's modified Eagle's medium (DMEM; Nacalai Tesque, Kyoto, Japan) supplemented with 10% fetal bovine serum (FBS; Biosera, Ringmer, UK), 50 mg·mL^−1^ penicillin, and streptomycin (regular medium) in a 5% CO_2_ incubator. For starvation treatment, cells were washed with PBS and incubated in amino acid‐free DMEM without serum (starvation medium). Tetracycline‐on (Tet‐on) cells were generated by lentiviral transduction with a pCW vector containing the single‐vector Tet‐on component. Gene editing was performed using the pSpCas9(BB)‐2A‐Puro (pX459) (Addgene plasmid 62988), and puromycin was replaced with EGFP. To generate *ATG14* KO cells, HeLa cells were transfected with pX459 GFP carrying the sgRNA expression cassette to target *ATG14* (CAGAGGCATAATCGCAAACT). On day 3 post‐transfection, GFP‐positive cells were sorted using an FACSAriaII flow cytometer and plated on a 96‐well plate. Single colonies were expanded into 24‐well plates before screening for depletion of *ATG14* by immunoblotting. For drug treatment, cells were incubated for the indicated times in medium containing one of the following reagents: 0.1 μm BafA (LC Laboratories), 5 μm MG132 (LifeSensors), 1 μm Torin1 (Tocris Bioscience), 50 μg·mL^−1^ CHX (Sigma‐Aldrich), or 1 μm Dox (Clonetech, Mountain view, CA, USA).

### siRNA knockdown experiments

Stealth RNAi oligonucleotides were used for siRNA experiments (Thermo Fisher Scientific, Waltham, MA, USA). The sequences used were as follows: human FIP200 siRNA sense, 5′‐GGAAATGTATGAAGTTGCCAAGAAA‐3′ and luciferase siRNA sense, 5′‐CGCGGTCGGTAAAGTTGTTCCATTT‐3′. HeLa cells were seeded onto a 12‐well plate at 50% confluency. siRNA transfections were performed with Lipofectamine RNAiMAX transfection reagent (Thermo Fisher Scientific) using 20 nm siRNA in Opti‐MEM (Gibco, Tokyo, Japan) with no antibiotic. At 48 h post‐transfection, cells were again transfected with the same siRNA and cultured for an additional 4 days before analysis.

### Lentiviral infections and generation of stable cell lines

Stable cell lines were generated using a lentiviral expression system. 293FT cells were transiently cotransfected with lentiviral vectors using PEI MAX reagent (Polyscience, Inc., Warrington, PA, USA). After culturing for 72 h, the growth medium containing lentivirus was collected. HeLa cells were incubated with the collected virus‐containing medium with 10 mg·mL^−1^ polybrene for 24 h. Uninfected cells were removed using 1 μg·mL^−1^ puromycin (InvivoGen, San Diego, CA, USA).

### Fluorescence microscopy

Cells expressing fluorescent‐tagged protein were grown on coverslips, fixed in 3.7% formaldehyde in PBS for 15 min, and observed under a fluorescence microscope (Delta vision Applied Precision, Issaquah, WA, USA) using a 60 × oil‐immersion objective lens with a numerical aperture (NA) of 1.42. For immunostaining, fixed cells were permeabilized with 50 μg·mL^−1^ digitonin in PBS for 5 min, blocked with 10% FBS in PBS for 30 min, and incubated with primary antibodies for 1 h. After washing, cells were incubated with Alexa Fluor 488/568/647‐conjugated goat anti‐rabbit or mouse IgG secondary antibodies (Thermo Fisher Scientific) for 1 h.

### Immunoblotting

Cells were washed with PBS and lysed in lysis buffer (1% Triton X‐100, 50 mm Tris/HCl pH 7.5, 1 mm EDTA, and 150 mm NaCl) supplemented with protease‐inhibitor cocktail (complete EDTA‐free protease inhibitor, Roche Diagnostics, Tokyo, Japan) and 1 mm phenylmethanesulfonyl fluoride (PMSF) for 15 min at 4 °C. The lysates were clarified by centrifugation at 20 000 ***g*** for 5 min and then 6 × SDS sample buffer was added. Samples were boiled for 5 min prior to SDS/polyacrylamide gel electrophoresis (SDS/PAGE). Ten micrograms of protein per lane were separated by SDS/PAGE and transferred to polyvinylidene difluoride membrane (Millipore, Billerica, MA, USA). Immunoblot analysis was performed with the indicated antibodies, and the immunoreactive proteins were visualized using ImmunoStar Zeta (Wako Pure Chemical Industries, Ltd.).

### Insolubility assay

Cells were washed with PBS and lysed in lysis buffer (0.5% Triton‐100, 0.5% deoxycholate, 50 mm Tris/HCl pH 7.5, 2 mm EDTA, and 150 mm NaCl) supplemented with protease‐inhibitor cocktail and 1 mm PMSF for 30 min at 4 °C. After centrifugation at 20 000 ***g*** for 20 min, the supernatant was reserved in a separate tube. The pellet was washed once with lysis buffer, recovered by centrifugation, and resuspended in the same volume of lysis buffer. The genomic DNA in the insoluble fraction was sheared by sonication. The soluble and insoluble fractions were denatured in sample buffer and analyzed by SDS/PAGE.

### Flow cytometry analysis

Cells were recovered by detachment from the dish with trypsin and EDTA, and sedimented by centrifugation at 3000 ***g*** for 2 min. The cells were passed through a 70‐μm cell‐strainer and resuspended in 10% FBS and 1 μg·mL^−1^ 4′,6‐diamidino‐2‐phenylindole (DAPI) in PBS for flow cytometry analysis using a CytoFLEX S flow cytometer equipped with NUV 375‐nm (for DAPI), 488‐nm (for GFP), and 561‐nm (for mCherry) lasers (Beckman Coulter, Inc., Brea, CA, USA). Dead cells were detected by DAPI staining.

### Photobleaching experiments

Bleaching experiments were performed using a confocal laser microscope (FV1000 IX81; Olympus, Tokyo, Japan) using a 60 × oil‐immersion objective lens with a numerical aperture (NA) of 1.42. Cells stably expressing fluorescent protein fusion proteins were placed on a glass bottom dish for 1 day before the bleaching experiment. During live imaging, the culture dish was mounted in a chamber (Tokai Hit Co., Shizuoka, Japan) to maintain incubation conditions at 37 °C. For fluorescence recovery after photobleaching (FRAP) analysis, a circular region of interest (ROI) was bleached with a 488‐nm (for GFP) or 543‐nm (for mCherry) laser at 100% power, and images were acquired every 60 s. For fluorescence loss in photobleaching (FLIP) analysis, an ROI was bleached at 100% laser power and imaged. This process was repeated at 60‐s intervals. Relative fluorescence intensity was calculated using the following formula (It − Nt)/(I0 − N0), where It is the mean fluorescence intensity in the ROI at a given time point, Nt is the fluorescence intensity at a nonbleached control region at a given time point, I0 is the initial (prebleaching) mean fluorescence intensity in the ROI, and N0 is the initial mean fluorescence intensity in a nonbleached control region.

## Results

### Aggregates in autophagy‐deficient cells contain ubiquitinated proteins

**Figure 1:**
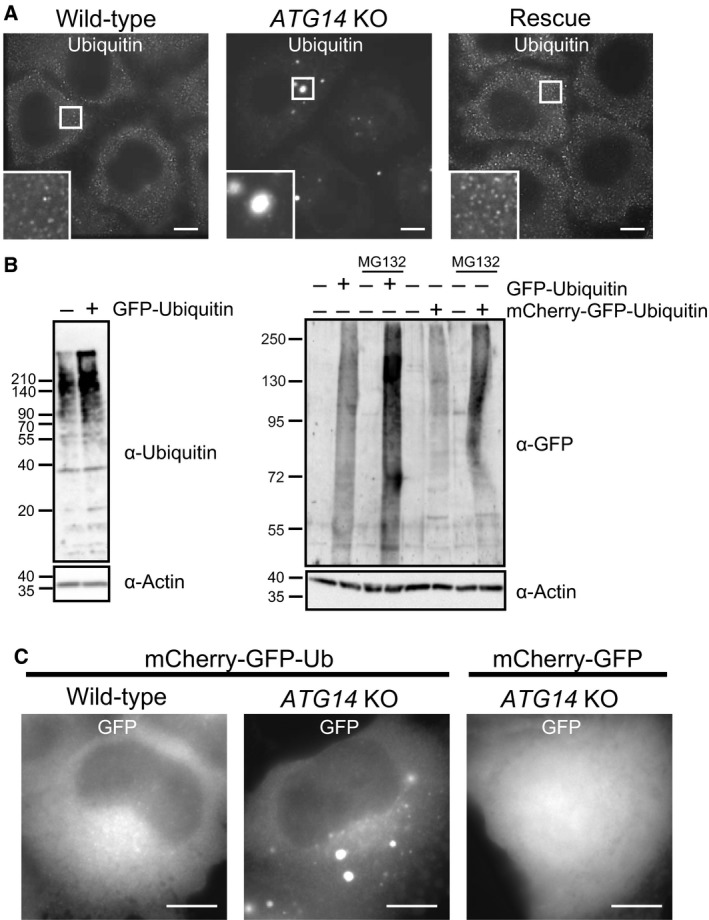
Ubiquitinated proteins form aggregates in autophagy‐deficient cells. (A) The endogenous ubiquitinated proteins in wild‐type cells, *Atg14* knockout (KO) cells, and *Atg14* KO cells expressing HA‐Atg14 rescue HeLa cells were analyzed by fluorescence microscopy using antibodies against polyubiquitin (FK2) and p62. A magnified image of the indicated region is shown in the inset. Scale bar, 5 μm. (B) HeLa cells stably expressing or not expressing GFP‐Ubiquitin or mCherry‐GFP‐Ubiquitin were cultured in the presence or absence of MG132 for 8 h and analyzed by immunoblotting using antibodies against ubiquitin, GFP, and β‐actin. (C) Wild‐type or *Atg14* KO HeLa cells stably expressing mCherry‐GFP or mCherry‐GFP‐Ubiquitin were analyzed by fluorescence microscopy.

Previous studies have demonstrated that defects in autophagy result in the accumulation of ubiquitin‐positive aggregates in various tissues 14, 24, 25, 26, 27. We confirmed that the cytosolic ubiquitin signal was decreased and that ubiquitin‐positive aggregates were formed *in vitro* in HeLa cells deficient in *ATG14* (Fig. 1A), which specifically functions in the early step in the autophagosome formation 28, 29. We confirmed that other autophagy protein‐deficient cell lines also showed similar aggregates (Fig. S1A). At least two possibilities might explain the accumulation of ubiquitinated aggregates in autophagy‐deficient cells: (a) the ubiquitinated proteins are direct substrates for basal autophagy, or (b) the accumulation of ubiquitinated aggregates is a secondary effect of a defective autophagy protein quality control system.

To investigate the lysosomal degradation of ubiquitinated proteins, we developed a novel fluorescent probe, mCherry‐GFP(C‐G)‐Ubiquitin (see next section for details). We first confirmed that the fluorescent protein tag on ubiquitin had no effect on ubiquitin conjugation. Immunoblots of fluorescent protein‐tagged ubiquitin showed the smear signals of high molecular weight proteins which increased by treatment with proteasome inhibitor (Fig. 1B). Furthermore, fluorescent protein‐tagged ubiquitin formed aggregates in *ATG14* KO cells but not wild‐type (WT) HeLa cells (Fig. 1B,C). In contrast, mCherry‐GFP (C‐G) without ubiquitin formed no aggregates even in *ATG14* KO cells (Fig. 1C). Polyubiquitinated GFP‐ubiquitin was rapidly degraded in a manner similar to that of polyubiquitinated HA‐Ubiquitin (Fig. S1B). These results suggest that the fluorescent protein‐tagged ubiquitin is incorporated in endogenous polyubiquitinated proteins.

### Ubiquitin is degraded by the proteasome, but not the lysosome

**Figure 2:**
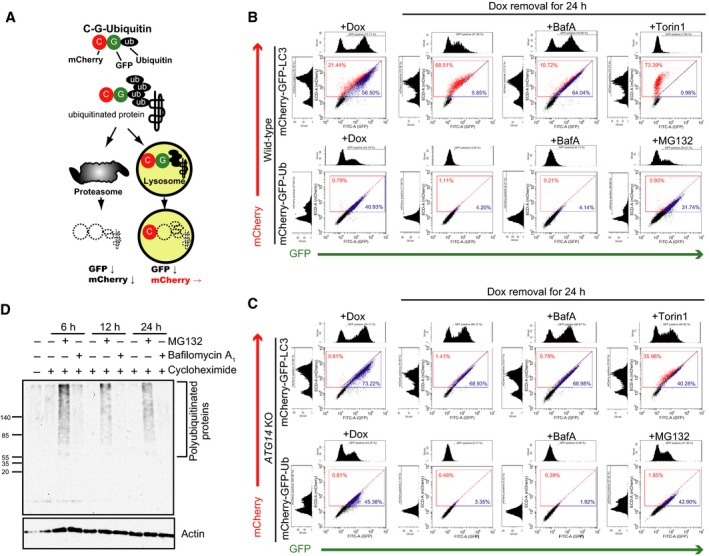
Suppression of autophagy does not affect the degradation of ubiquitinated proteins. (A) Schematic diagram of ubiquitinated protein degradation. (B,C) Wild‐type or *Atg14* KO HeLa tetracycline‐on (Tet‐on) cells expressing mCherry‐GFP‐Ubiquitin (Ub) or ‐LC3 were cultured in the presence of doxycycline (Dox). After removal of Dox, the cells were treated with MG132 or bafilomycinA_1_ (BafA) for 24 h and analyzed by flow cytometry. Histogram analysis and representative dot plots of green fluorescent protein (GFP) versus mCherry fluorescence intensities are shown. (D) HeLa cells were cultured in the presence or absence of cycloheximide with or without MG132, and bafilomycin A_1_ for the indicated times. Turnover of the endogenous ubiquitinated proteins was analyzed by immunoblotting using antibodies against ubiquitin and β‐actin.

In cells expressing C‐G‐Ubiquitin, the C‐G‐Ubiquitin is incorporated in the ubiquitin chain on endogenous proteins and then degraded by proteasomes or lysosomes via autophagy. Proteasomes degrade the full length of C‐G‐Ubiquitin, whereas lysosomes primarily degrade GFP (which is sensitive to acids) but not mCherry (which is insensitive to pH and lysosomal proteases) 30, 31. Therefore, transport of C‐G‐Ubiquitin into the lysosome changes the fluorescence ratio between GFP and mCherry, whereas proteasomal degradation does not (Fig. 2A). To monitor basal autophagy, C‐G‐Ubiquitin was expressed using the pulse‐chase system in Tet‐on cells.

mCherry‐GFP tandem fluorescent‐tagged LC3 (C‐G‐LC3) was used as a positive control for lysosomal degradation. Dox was removed from the cells and the cells were cultured for 24 h before flow cytometry analysis. As expected, C‐G‐LC3 was transported by basal autophagy into the lysosome where the GFP signal decreased, but the mCherry signal remained (Fig. 2B). In contrast, C‐G without LC3 showed only a mild reduction in both signals (Fig. S2A). Furthermore, autophagy induction by the mTor inhibitor, Torin1, facilitated the reduction of GFP fluorescence, but had little effect on mCherry fluorescence. Moreover, treatment with a lysosome inhibitor, bafilomycin A_1_, suppressed the reduction of the GFP signal (Fig. 2B). *ATG14* KO abolished the lysosomal degradation of C‐G‐LC3 (Fig. 2C). Next, we performed the same experiments with C‐G‐Ubiquitin. The results showed that the C‐G‐Ubiquitin signal was markedly diminished after Dox removal for 24 h, and this degradation was efficiently repressed by MG132, but not bafilomycin A_1_ (Fig. 2B). *ATG14* KO cells showed no change in the fluorescence ratio of C‐G‐Ubiquitin compared with that in WT cells (Fig. 2C). Similar results were obtained from autophagy knockdown cells using siRNA against FIP200 (Fig. S3A). In addition, the degradation of fluorescent‐tagged ubiquitin was delayed by the introduction of the K48R mutation in ubiquitin, but not the K63R mutation (Fig. S3B). These data indicate that autophagy does not contribute to the degradation of ubiquitinated proteins at the basal level. When cells were treated with the autophagy inducer Torin1, C‐G‐Ubiquitin was slightly degraded by lysosomes dependently of *ATG14* (Fig. S2B). C‐G without ubiquitin was also slightly degraded in the presence of Torin1 (Fig. S2A), indicating that some of the cytosolic C‐G‐Ubiquitin was transported into lysosomes by bulk autophagy.

We next examined the degradation of endogenous ubiuquitinated proteins using CHX chase experiments, which measure the half‐life of proteins in the presence of the translation inhibitor. The ubiquitinated proteins were significantly reduced after CHX treatment for 12 h and this reduction was inhibited by cotreatment with MG132 but not bafilomycinA_1_ (Fig. 2D). It is also known that CHX suppresses autophagy 32. Taken together, these results suggest that the ubiquitinated proteins are not major substrates for basal autophagy.

### Autophagosomes do not contain ubiquitinated proteins under basal conditions

**Figure 3:**
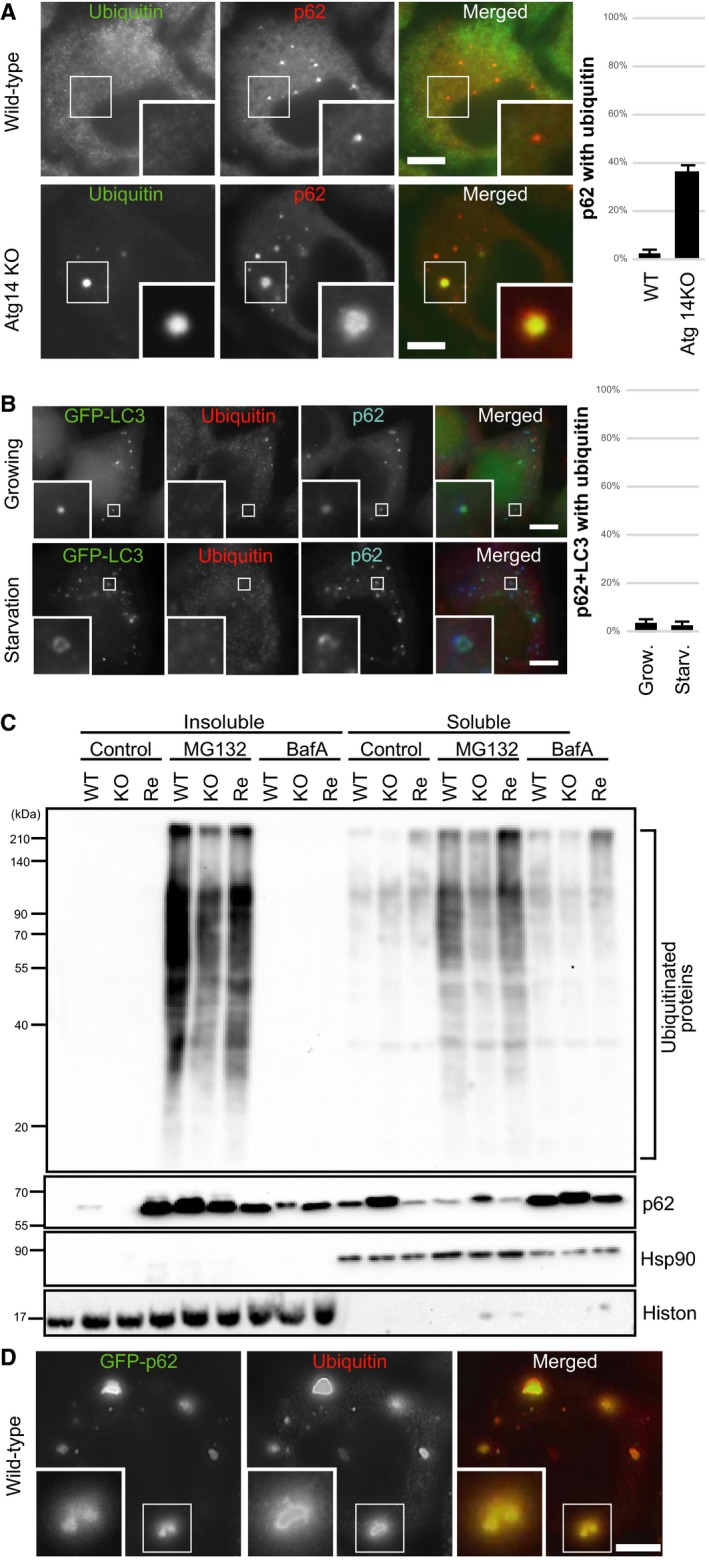
Autophagosomes do not contain ubiquitinated proteins. (A) Wild‐type or *Atg14* knockout (KO) HeLa cells were fixed and stained with anti‐ubiquitin (FK2) and anti‐p62 antibodies, and analyzed by immunofluorescence microscopy. Quantification of ubiquitin positivity (%) of the p62 puncta is shown. Data represent mean ± SE of five images. (B) Wild‐type HeLa cells stably expressing GFP‐LC3 were fixed and stained with anti‐ubiquitin (FK2) and anti‐p62, then analyzed by immunofluorescence microscopy. Quantification of ubiquitin positivity (%) of the p62+LC3 puncta is shown. Data represent mean ± SE of five images. (C) The endogenous ubiquitinated proteins in wild‐type cells (WT), *Atg14* KO cells (KO), and *Atg14* KO cells expressing HA‐Atg14 rescue HeLa cells (Re) were analyzed by immunoblotting using antibodies against ubiquitin (FK2), p62, Hsp90 (soluble fraction marker), and Histon H3 (insoluble fraction marker). (D) Wild‐type HeLa cells stably overexpressing GFP‐p62 were fixed and stained with anti‐ubiquitin (FK2) and analyzed by immunofluorescence microscopy.

As we detected no lysosomal degradation of ubiquitin under basal conditions, we suspected that the autophagosomes do not engulf polyubiquitinated proteins. Staining of endogenous ubiquitinated proteins in *ATG14* KO cells using antibodies against polyubiquitin and p62 (a selective substrate for autophagy) showed that polyubiquitin‐positive aggregates in autophagy‐deficient cells were colocalized with p62. In contrast, polyubiquitinated proteins in WT cells were not observed in the p62‐positive autophagosomes under basal conditions (Fig. 3A). Even with starvation‐induced autophagy, polyubiquitin was not detectable in LC3‐positive autophagosomes (Fig. 3B). Next, we investigated whether the total amount of ubiquitinated proteins increased in autophagy KO cells. Proteasome inhibitor treatment increased ubiquitinated proteins in the both soluble and insoluble fractions 33, 34 (Fig. 3C). Whereas autophagy KO cells showed marked accumulation of p62, the KO cells showed no apparent accumulation of polyubiquitinated proteins in the total, soluble or insoluble fractions (Figs 3C and S1C). Bafilomycin A_1_ treatment for 24 h increased the levels of p62 but not polyubiquitinated proteins (Fig. 3C). We speculated that p62 might trap general ubiquitinated proteins in autophagy KO cells despite no apparent increase in ubiquitinated proteins. Indeed, overexpression of GFP‐p62 in WT cells caused aggregation with polyubiquitinated proteins (Fig. 3D). These data suggest that autophagosomes do not constitutively engulf polyubiquitinated proteins.

### Soluble ubiquitinated proteins are trapped within p62‐positive aggregates

**Figure 4:**
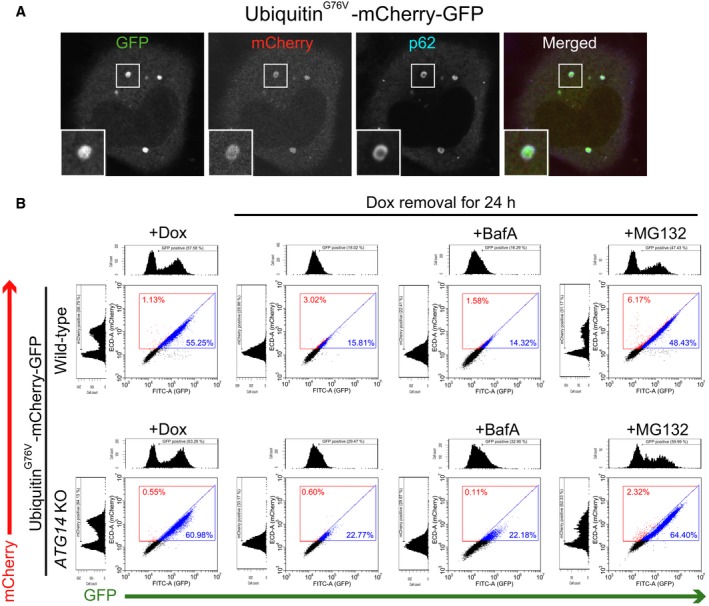
Ubiquitinated soluble proteins are trapped in aggregates but not degraded by autophagy. (A) *Atg14* KO HeLa Tet‐on cells expressing Ubiquitin^G76V^‐mCherry‐GFP were fixed and stained with anti‐p62 antibody and analyzed by fluorescence microscopy. Scale bar, 5 μm (inset). (B) Wild‐type or *Atg14* KO HeLa Tet‐on cells expressing ubiquitin^G76V^‐mCherry‐GFP were cultured in the presence of Dox. After removal of Dox, the cells were treated with MG132 or BafA for 24 h and analyzed by flow cytometry. Histogram analysis and representative dot plots of GFP fluorescence intensity versus mCherry fluorescence intensity are shown.

As p62 aggregates incorporate general ubiquitinated proteins, we investigated whether soluble ubiquitinated proteins could be targeted to p62 aggregates. GFP is a soluble and stable protein in cells. When uncleaved ubiquitin fused at the N terminus of GFP, the Ub^G76V^‐GFP is rapidly polyubiquitinated and degraded by proteasomes 35. We generated cells that stably expressed Ub^G76V^‐C‐G under the control of the Tet‐on system and observed its localization in *ATG14* KO cells. Ub^G76V^‐C‐G but not C‐G was localized in the large aggregates with p62 in *Atg14* KO cells (Figs 1C and 4A), indicating that soluble ubiquitinated protein is trapped in p62 aggregates. Consistent with a previous report that excess p62 in autophagy KO cells delays ubiquitinated protein delivery to the proteasomes 36, flow cytometry analysis showed that the degradation of Ub^G76V^‐C‐G was slightly delayed in autophagy KO cells compared with WT cells (Fig. 4B). However, efficient lysosomal degradation of Ub^G76V^‐C‐G was not detected, and the degradation was completely dependent on proteasomes (Fig. 4B). Amount of the lysosomal degradation of Ub^G76V^‐C‐G was similar to C‐G which is degraded by nonselective autophagy (Fig. S2A). These results suggest that p62 aggregates can target soluble ubiquitinated proteins in a selective autophagy‐independent manner.

### Ubiquitinated proteins in aggregates are mobile in autophagy‐deficient cells

**Figure 5:**
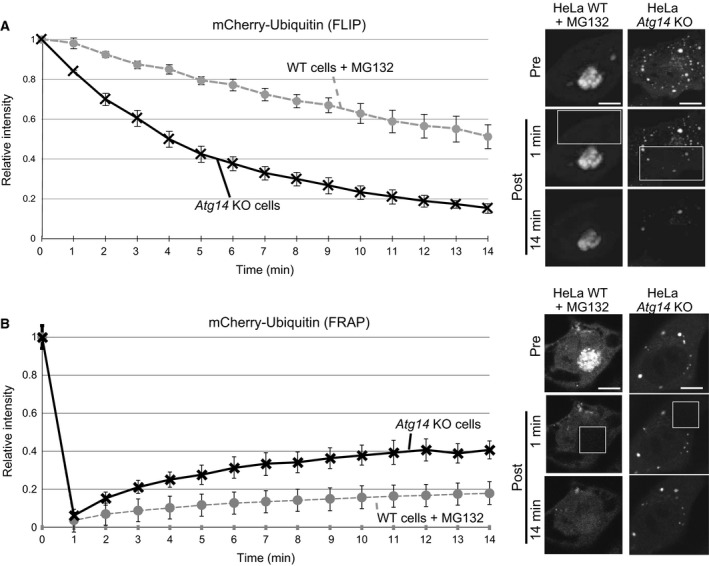
Ubiquitinated proteins in aggregates of autophagy‐deficient cells are mobile. (A,B) Mobility analysis of ubiquitin aggregates. Wild‐type (WT) or *Atg14* KO HeLa cells coexpressing mCherry‐Ubiquitin and GFP‐p62 were cultured in the presence or absence of MG132 for 24 h and subjected to fluorescence loss in photobleaching (FLIP) (A) and fluorescence recovery after photobleaching (FRAP) (B) analysis. Fluorescence intensities of the aggregates were quantitatively analyzed before and during recovery after bleaching of a cytoplasmic area (A) or before and after bleaching of an aggregate (B). The data shown represent the average ± SE from at least three independent experiments. Representative confocal images are shown. The box on the image marks the area of bleaching.

We hypothesized that if ubiquitin‐positive aggregates in autophagy KO cells consist mainly of soluble ubiquitinated proteins trapped by p62, the aggregates would not be static. Therefore, we further characterized the ubiquitinated aggregates in autophagy‐deficient cells using FLIP and FRAP assays. We bleached a region of mCherry‐positive cytoplasm and then recorded the subsequent fluorescence over 14 min (Fig. 5A). We found that the fluorescence of ubiquitin‐positive aggregates in *ATG14* KO cells diminished faster than that of aggregates in MG132‐treated WT cells. Consistently, when a region of mCherry‐Ubiquitin‐positive aggregates was bleached, more mCherry‐ubiquitin fluorescence was recovered in *ATG14* KO cells compared to that recovered in MG132‐treated WT cells (Fig. 5B). We further investigated the mobility of ubiquitin and p62 in aggregates induced by proteasome inhibition or autophagy deficiency (Fig. S4). Although both ubiquitin and p62 showed similar kinetics in MG132‐induced aggregates, ubiquitin showed faster mobility than p62 in aggregates of autophagy‐deficient cells. These data suggest that ubiquitinated proteins in aggregates are mobile in autophagy‐deficient cells.

## Discussion

It is well known that ubiquitin‐positive aggregates accumulate in the tissues of autophagy KO mice 13, 14, 37, 38. In this study, we investigated the relationship between ubiquitinated proteins and autophagy by analyzing the degradation pathways of ubiquitinated proteins under basal conditions using a novel fluorescence ratiometric assay for ubiquitin with a pulse‐chase system. Using this assay, we revealed that ubiquitinated proteins are not degraded by autophagy under basal conditions (Fig. 2). If ubiquitinated proteins were major substrates for basal autophagy, the amount of ubiquitinated proteins should be increased in autophagy‐deficient cultured cells. However, we detected no significant accumulation of ubiquitinated proteins in autophagy KO cells by western blotting (Fig. 3C); furthermore, no polyubiquitinated proteins were observed in autophagosomes (Fig. 3A). These results suggested that ubiquitinated proteins are not the main substrates for basal autophagy.

Our results suggest that increased total ubiquitinated protein in the autophagy‐deficient liver is not due to a defect in the degradation of ubiquitinated proteins, but might instead be due mainly to Nrf2 activation associated with p62 accumulation, which leads to hepatocellular carcinoma 16, 27, 39, 40. On the other hand, autophagy‐deficient cultured cells showed p62 and ubiquitin‐positive aggregates without an increase in the total amount of ubiquitinated proteins (Fig. 3C). It is thought that general ubiquitinated proteins might be trapped in aggregates by p62. Our results support this view, as overexpression of p62 formed aggregates with ubiquitinated proteins even in wild‐type cells (Fig. 3D). Notably, ubiquitin‐positive inclusions were dispersed by the loss of p62 in autophagy‐deficient neurons 39, 41. Ubiquitinated soluble protein (fluorescent protein) but not soluble protein without ubiquitin was also incorporated in the aggregates in *ATG14* KO cells (Figs 1C and 4A). Hence, p62‐positive aggregates in autophagy KO cells trap any ubiquitinated proteins in the cytoplasm. Indeed, no specific polyubiquitin chains were accumulated in autophagy KO mice 16.

Although ubiquitin is an abundant protein (constituting ~ 1–2% of total protein) and is considered to be stable based on its compact and globular structure, the total amount of free ubiquitin is regulated, and the disruption of ubiquitin homeostasis induces a stress response 42. Our data showed that ubiquitin is undetectable after inhibition of synthesis for 1 day, suggesting that the half‐life is approximately half a day in HeLa cells (Fig. 2). Several reports have suggested that the half‐life of ubiquitin in mammalian cells is 28–31 h in lung fibroblasts, 9 h in leukemia cells, and 18 h in HeLa cells 22, 43, 44. These variations may be due to differences in cell type, growth speed, and experimental design. At least, these data indicate that ubiquitin is not a long‐lived protein. Although both proteasomal and lysosomal degradation pathways have been described to be involved in ubiquitin degradation 20, 22, it is unclear which pathway predominates. Although the ubiquitin chain on ubiquitinated protein is deubiquitinated by deubiquitinating enzymes, such as Ubp6 and Rpn11 on the proteasome and recycled to the cytosol, ubiquitin at the most proximal part of the polyubiquitin chain is degraded along with the substrate 20, 45. It is also reported that lysosomes also involves in ubiquitin degradation, and ubiquitin colocalizes with lysosomes in the cell 46, 47. Moreover, there is report that starvation accelerates ubiquitin degradation 22. Our quantitative measurement of ubiquitin degradation revealed that autophagy KO and lysosomal inhibitors had a little effect on ubiquitin metabolism under basal conditions, and only a low level of degradation of ubiquitinated proteins was observed in lysosomes when autophagy was induced by an mTor inhibitor (Fig. S2B). However, we also observed similar lysosomal degradation of fluorescent protein alone without ubiquitin in the presence of the mTor inhibitor (Fig. S2A). These data suggest that the selective autophagic degradation of ubiquitinated proteins, at least under basal and starved conditions, is a very minor pathway; a portion of them might be degraded by bulk autophagy.

Recent studies revealed that ubiquitination of selective autophagic substrates such as mitochondria and aggregated proteins is a critical step for their recognition by autophagosomes. Indeed, most autophagy adaptors (e.g., p62, OPTN, NDP52, and NBR1), which link the substrate with LC3, contain ubiquitin‐binding domains. Given that autophagy KO mice accumulate ubiquitinated proteins, it was speculated that cytosolic ubiquitinated proteins would be targets for basal autophagy. However, our data do not support the hypothesis that ubiquitinated proteins are direct substrates for basal autophagy. In addition, no reports have shown the accumulation of ubiquitinated proteins in autophagy‐deficient yeast. Therefore, aggrephagy of ubiquitinated proteins might not occur under basal conditions, but rather, it is induced under specific conditions such as the accumulation of mutated protein.

The critical substrate of basal autophagy as a protein quality control system remains unclear. One possibility is that basal autophagy nonselectively degrades cytosolic proteins and is important for protein homeostasis 13. When autophagy is suppressed, cytosolic proteins that were slated for degradation by autophagy accumulate damage(s). Then, the damaged proteins are secondarily ubiquitinated to form aggregates with p62, thereby affecting cell homeostasis. Another possible mechanism is ubiquitin‐independent selective autophagy 48. For example, ferritinophagy, which supplies iron by autophagic degradation of ferritin; this process might have a critical role in neurons 49. Indeed, ferritin clusters accumulate independently on p62 in autophagy KO cells, and autophagy KO mice develop iron deficiency 27, 50. Furthermore, a mutation in an autophagy gene, WIPI4, which causes static encephalopathy of childhood with neurodegeneration in adulthood, is characterized as a subtype of neurodegeneration with brain iron accumulation 51. Recent proteomics analysis demonstrated that the proteasome and CCT/TRiC chaperonin complex are substrates of basal autophagy; however, the role of degradation by basal autophagy is unclear 17. Further studies will be required to elucidate the precise mechanisms of basal autophagy and their roles in neurodegenerative diseases.

## Author contributions

KT and EI designed the project. KT carried out the experiments and analyzed the data. KT, AM, and EI wrote the manuscript.

## Supporting information


**Fig. S1.** Ubiquitinated protein aggregates in autophagy‐deficient cells.
**Fig. S2.** Analysis of C‐G‐Ubiquitin and mCherry‐GFP with Torin1.
**Fig. S3.** Flow cytometry analysis of knockdown of FIP200 and ubiquitin mutants.
**Fig. S4.** FLIP analysis of ubiquitin together with p62.
