# Supplementary material for: Dissection of ubiquitinated protein degradation by basal autophagy
Source: FEBS Lett. 2017 Apr 18;591(9):1199–211. doi: 10.1002/1873-3468.12641 (PMC5435929; doi:10.1002/1873-3468.12641)
Supplement: Supplementary file 1 — Fig. S1. Ubiquitinated protein aggregates in autophagy‐deficient cells. Fig. S2. Analysis of C‐G‐Ubiquitin and mCherry‐GFP with Torin1. Fig. S3. Flow cytometry analysis of knockdown of FIP200 and ubiquitin mutants. Fig. S4. FLIP analysis of ubiquitin together with p62. [file FEB2-591-1199-s001.pdf]

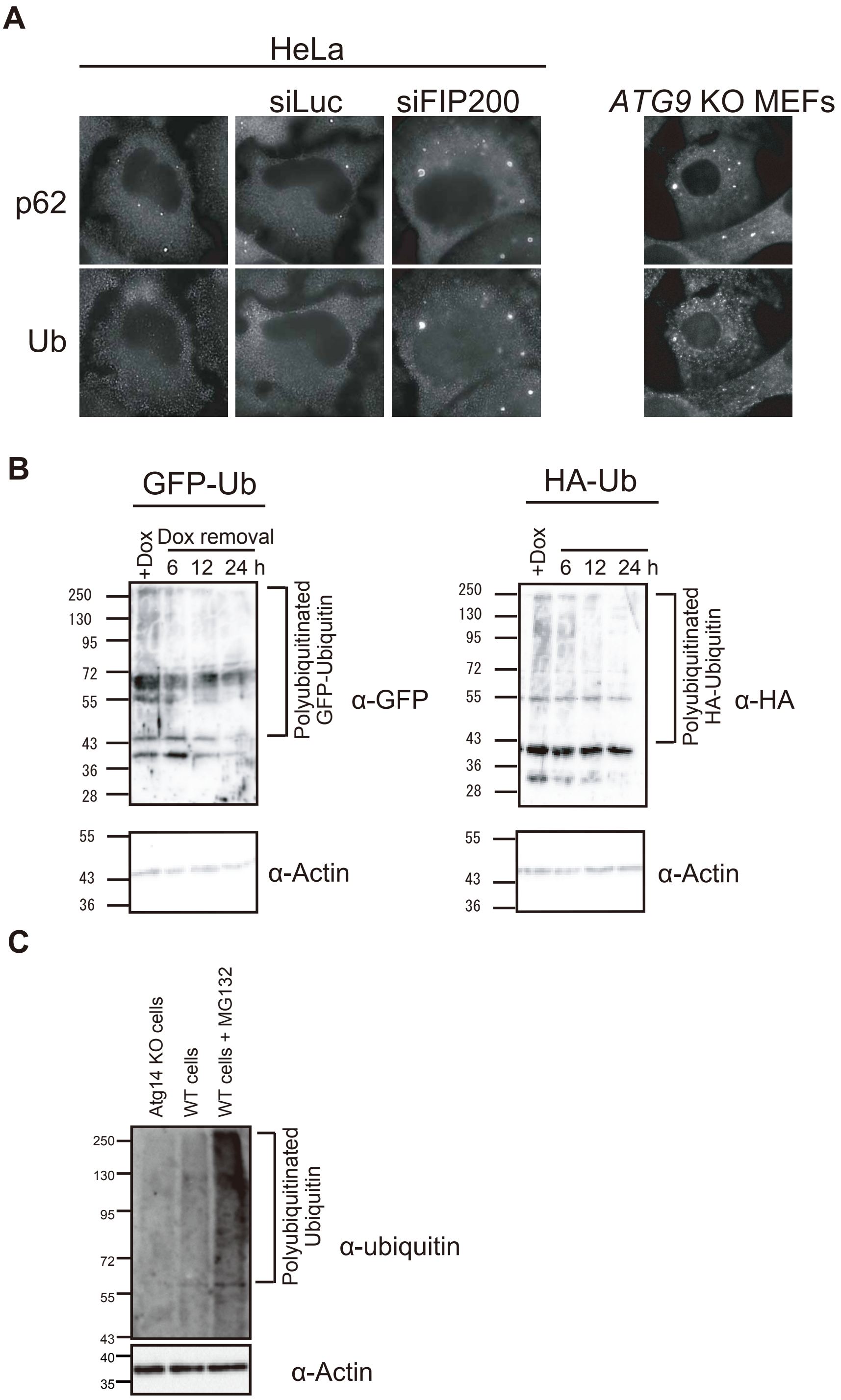

Figure S1. Ubiquitinated protein aggregates in autophagy-deficient cells

(A) Wild-type HeLa cells were treated with FIP200 siRNA or control Luciferase siRNA twice in 5 days, fixed, stained with anti -ubiquitin (FK2) and anti -p62 antibodies, and analyzed by immunofluorescence microscopy. *Atg9* KO MEF cells were subjected to the same analysis. Scale bar, 5 μm (inset). (B) HeLa cells stably expressing GFP -Ubiquitin or HA -Ubiquitin were analyzed by immunoblotting using antibodies against GFP, HA, and β-actin. (C) HeLa cells were treated with or without MG132 for 8 h and analyzed by immunoblotting using antibodies against ubiquitin and β-actin.

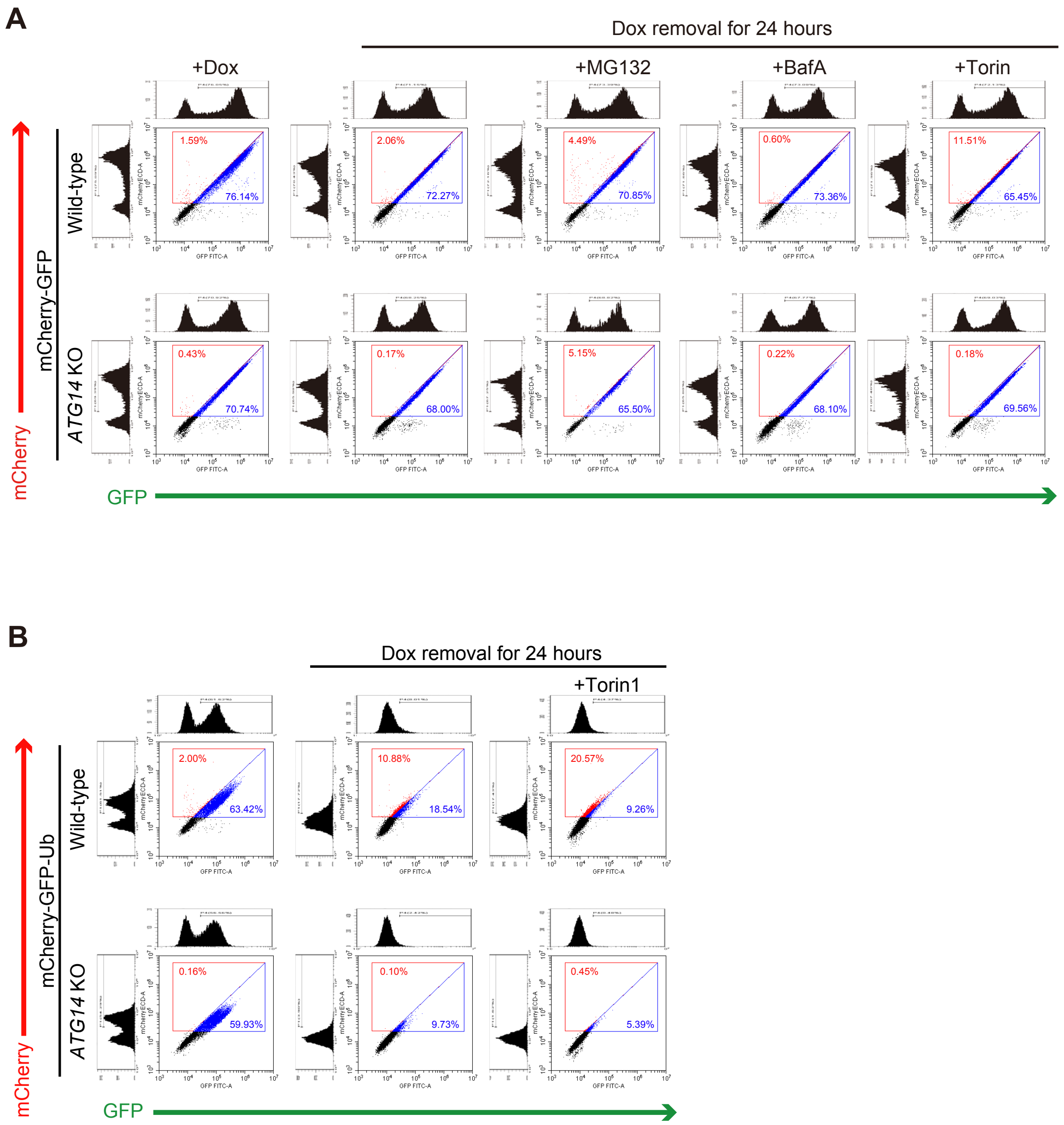

Figure S2. Analysis of C-G-Ubiquitin and mCherry-GFP with Torin1

(A) Wild-type or *Atg14* KO HeLa Tet-on cells expressing mCherry-GFP were cultured in the presence of Dox. After removal of Dox, the cells were treated with MG132, BafA or Torin1 for 24 h and analyzed by flow cytometry. Histogram analysis and representative dot plots of GFP fluorescence intensity versus mCherry fluorescence are shown. (B) Wild-type or *Atg14* KO HeLa Tet-on cells expressing mCherry-GFP-Ubiquitin (Ub) were cultured in the presence of Dox. After removal of Dox, the cells were treated with Torin1 for 24 h and analyzed by flow cytometry. Histogram analysis and representative dot plots of GFP fluorescence intensity versus mCherry fluorescence intensity are shown.

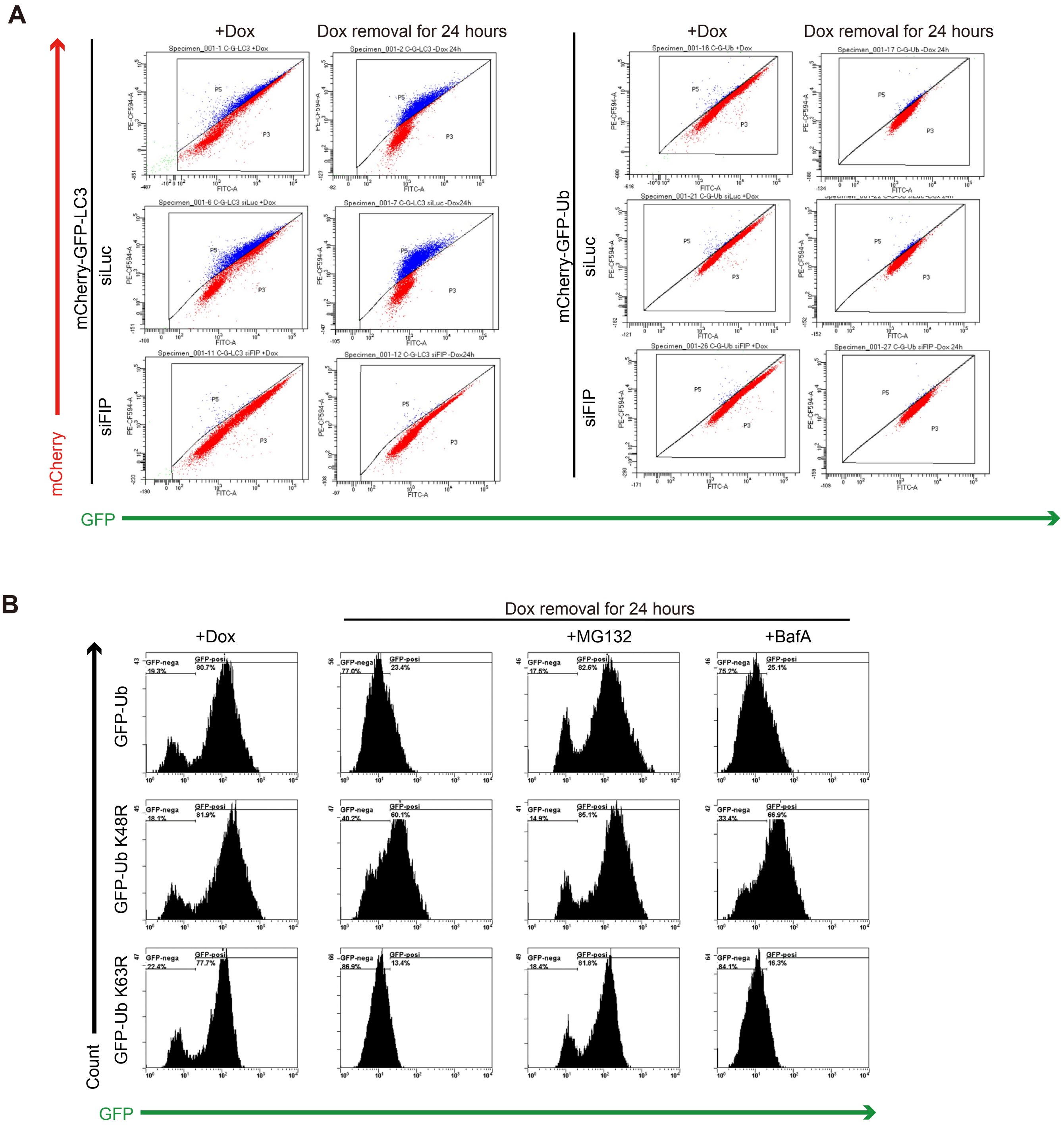

Figure S3. Flow cytometry analysis of knockdown of FIP200 and ubiquitin mutants

(A) Wild-type HeLa cells were treated with FIP200 siRNA or control Luciferase siRNA twice in 5 days, cultured in the presence of Dox. After removal of Dox for 24 h, cells were analyzed by flow cytometry. Representative dot plots of GFP fluorescence intensity versus mCherry fluorescence intensity are shown. (B) Wild-type HeLa Tet-on cells expressing GFP-Ubiquitin WT, K48R or K63R were cultured in the presence of Dox. After removal of Dox, the cells were treated with MG132 or BafA for 24 h and analyzed by flow cytometry. Representative histograms of GFP are shown.

A

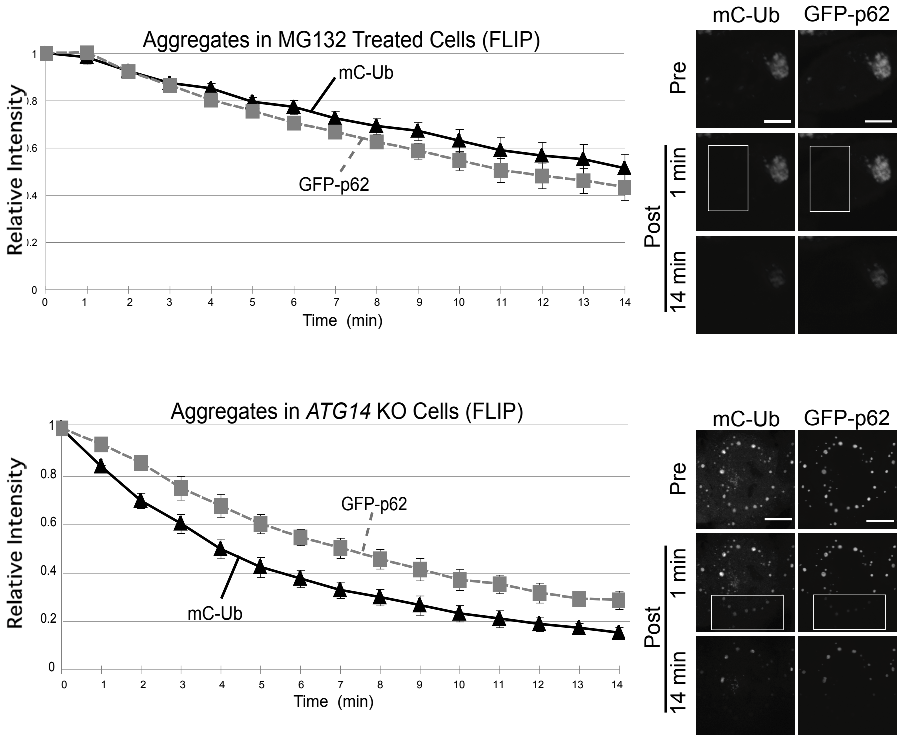

Figure S4. FLIP analysis of ubiquitin together with p62

(A) Mobility analysis of ubiquitin aggregates. Wild-type or *Atg14* KO HeLa cells co-expressing mCherry-Ubiquitin and GFP-p62 cultured in the presence or absence of MG132 for 24 h and were subjected to FLIP analysis.
